# The emerging roles of ubiquitin-like modifications in regulating HIV replication and host defense

**DOI:** 10.3389/fcimb.2025.1593445

**Published:** 2025-06-11

**Authors:** Jinsong Yuan, Huihan Wang, Xiaodong Sun, Chen Huan

**Affiliations:** ^1^ Center of Infectious Diseases and Pathogen Biology, Institute of Virology and AIDS Research, Key Laboratory of Organ Regeneration and Transplantation of The Ministry of Education, The First Hospital of Jilin University, Changchun, Jilin, China; ^2^ Department of Hepatobiliary and Pancreatic Surgery, General Surgery Center, The First Hospital of Jilin University, Changchun, Jilin, China

**Keywords:** UBLs, HIV-1, SUMOylation, ISGylation, NEDDylation, ATG8ylation, FAT10ylation

## Abstract

As a post-translational modification (PTM) mechanism analogous to ubiquitination, ubiquitin-like (UBL) modification plays a crucial regulatory role in virus-host interactions. With the increasing discovery of UBL modification types, their roles in diverse biological process, including HIV infection, have gained growing attention. Rather than merely serving as anti-HIV defenses or being exploited by the virus, UBLs often exert dual roles by modulating both host restriction factors and viral proteins, thereby impacting key steps of HIV life cycle, immune evasion, and intracellular signaling. This article summarizes recent advances on the contribution of UBLs in regulating HIV replication and host defense, highlighting their indispensable roles in arms races between HIV and host, aiming to provide a theoretical framework for developing novel therapeutic strategies against HIV-1 targeting virus-host interactions.

## 1 Introduction

Acquired immunodeficiency syndrome (AIDS) is an immune deficiency disorder caused by human immunodeficiency virus (HIV) infection (Saunders et al., 2024; Haynes et al., 2016; Chen et al., 2022; Guo et al., 2021b). HIV impairs immune defense by infecting CD4^+^ T cells, leading to severe immune dysfunction. According to UNAIDS estimates, approximately 39 million people worldwide were living with HIV by the end of 2022. Despite the widespread use of antiretroviral therapy (ART), HIV remains a major global public health challenge, particularly in resource-limited regions (Board et al., 2022; Matsui and Miura, 2023; Sheykhhasan et al., 2021; Ouyang et al., 2024). In the evolutionary arms race between HIV and host, the host employs various defense strategies. Restriction factors function by directly blocking specific steps of the viral life cycle, whereas immune recognition mechanisms detect viral signatures and trigger both initiate innate and adaptive immune responses. In return, HIV-1 employs strategies such as high mutation rates, hijacking PTM systems to antagonize host factors and evade restriction. This persistent conflict drives HIV’s adaptive evolution while compelling the host to refine defense strategies to suppress viral persistence. Both HIV-1 and host continuously optimize their strategy to achieve evolutionary advantages (Oswald et al., 2023).

Ubiquitin is a 76-amino-acid small protein ubiquitously expressed in all eukaryotic cells, with nearly all cellular proteins being subject to ubiquitination (Swatek and Komander, 2016). As a prevalent PTM, ubiquitination relies on a cascade of enzymatic catalysis mediated by E1 ubiquitin-activating enzymes (Schulman and Harper, 2009), E2 ubiquitin-conjugating enzymes (Ye and Rape, 2009), and E3 ubiquitin ligases (Deshaies and Joazeiro, 2009; Smit and Sixma, 2014; Rotin and Kumar, 2009). Mechanistically, the E1 enzyme catalyzes ATP-dependent Ub activation, forming a Ub-AMP intermediate that is subsequently transferred to E2 via a thioester bond. Then E2 cooperates with E3 ligases, which specifically recognize and bind substrate proteins, enabling Ub transfer from E2 to lysine residues on target substrates. Canonical ubiquitination regulates fundamental biological processes including protein degradation, signal transduction, cell cycle control, and immune responses (Reichard et al., 2016; Smith et al., 2017; Dang et al., 2021; Liu et al., 2024). Notably, this modification machinery serves not only as a core cellular regulatory mechanism but also as a critical battlefield in virus-host arms races. During co-evolution with hosts, multiple viruses have developed strategies to hijack the host ubiquitination system, thereby remodeling the cellular microenvironment and achieving immune evasion. A classic example is HIV-1 Vif protein, which recruits host ubiquitination components to mediate degradation of the antiviral restriction factor APOBEC3G, effectively escape from host restriction (Albin and Harris, 2010; Wolf and Goff, 2008).

**Figure 1:**
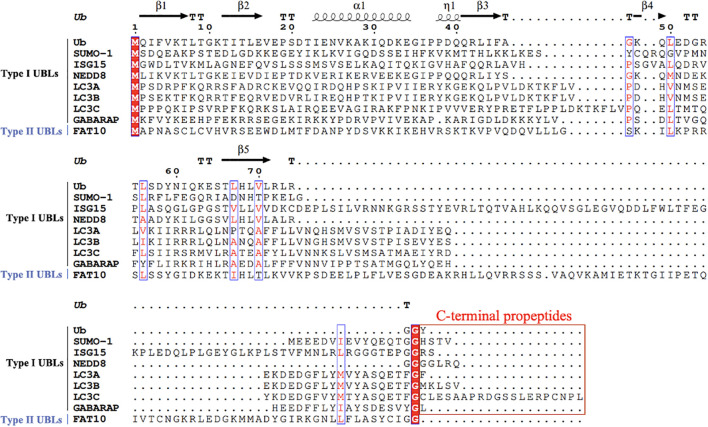
Multiple sequence alignment of Ub and UBLs. A multiple sequence alignment of Ub and various UBLs was performed using ClustalW. The residue numbering is based on Ub’s first methionine (M1). UBLs are classified into two types: Type I UBLs (e.g., SUMO-1, ISG15, NEDD8, LC3A, LC3B, LC3C, GABARAP), which require the removal of C-terminal propeptides prior to functional activation, and Type II UBLs (e.g., FAT10), which do not require such processing. Conserved residues are shown in red font and enclosed in blue boxes. Functionally important C-terminal glycine residues are also indicated. C-terminal propeptides regions in Type I UBLs are enclosed in red boxes to highlight their required removal before activation. Gaps introduced for optimal alignment are represented by dots. Ub residues are numbered every 10 amino acids along the alignment to assist in identifying conserved and functionally relevant regions. Secondary structure elements of Ub (β-strands, α-helix, and the η-helix) are annotated above the alignment, based on the crystal structure of ubiquitin.

Recent studies have revealed a series of UBLs including small ubiquitin-like modifiers (SUMOs), interferon-stimulated gene 15 (ISG15), developmentally down-regulated 8 (NEDD8), autophagy-related protein 8 (ATG8), and human leukocyte antigen (HLA)-F adjacent transcript 10 (FAT10) that share structural homology with Ub. Both Ub and UBLs utilize exposed C-terminal glycine residues for substrate conjugation. Sequence alignment (**Figure 1**) categorizes UBLs into two classes: Type I UBLs, which are precursor forms requiring proteolytic activation to expose the reactive glycine (e.g., SUMOs, ISG15, NEDD8, and ATG8/LC3-GABARAP family), and Type II UBLs, which are constitutively active forms with intrinsically exposed glycine (e.g., FAT10). With the exception of FAT10ylation (which lacks identified E3 ligases), all UBLs employ the conserved E1-E2-E3 enzymatic cascade. Crucially, each UBL system orchestrates specialized regulatory functions through unique enzymatic networks: SUMOylation dynamically modulates protein interaction and subcellular trafficking (Han et al., 2018); ISG15ylation exhibits dual roles as covalent modifiers or free molecules to antagonize ubiquitination during antiviral responses (Okumura et al., 2008; Lenschow et al., 2007); NEDDylation regulates substrate stability, conformational dynamics, and functional activation (Zhao et al., 2014); ATG8ylation engages its N-terminal α-helical domain to mediate selective autophagic degradation of membrane-associated targets (Zhang et al., 2023; Deretic et al., 2024); while FAT10ylation coordinates immune homeostasis through inducing proteasomal degradation of substrate (Schmidtke et al., 2009; Bialas et al., 2015).

Compared to classical PTMs such as phosphorylation, acetylation, and methylation (Bilbrough et al., 2022; Ferrante et al., 2020), UBLs can form mono- or polymeric chains through diverse linkage types, generating highly complex signaling codes that enable more precise regulation of cellular responses. During HIV infection, UBL modifications play dual roles by regulating both viral protein functions and host immune responses, exhibiting greater specificity and immune evasion capabilities in the interactions between the virus and the host. This review summarizes current research advancements on UBLs, with particular focus on their functions during HIV infection. Our synthesis aims to provide a theoretical basis for developing potential targeted therapeutics and offers predictive insights into future research directions.

## 2 SUMOylation

### 2.1 Introduction of SUMOylation

The SUMO molecule has a molecular weight of approximately 11 kDa and contains a characteristic βββαβαβ fold with a C-terminal diglycine motif (Wang and Dasso, 2009). Its three-dimensional structure resembles Ub, yet distinct surface charge distribution and amino acid sequences confer divergent functional properties. SUMO requires specific cleavage of the C-terminal propeptides by ubiquitin-like specific protease 1 (ULP1) or sentrin-specific protease 1 (SENP) (Nayak and Müller, 2014). SUMOylation covalently conjugates SUMO molecules to lysine residues on substrates through an enzymatic cascade involving E1 (SAE1/SAE2), E2 (Ubc9), and E3 ligases (PIAS family, RanBP2, etc.). Notably, the SUMOylation not only mediates covalent modification of SUMO molecule to the lysine residues of substrates via isopeptide bonds, but also facilitates non-covalent interactions with effector proteins through SUMO-interacting motifs (SIMs) (Minty et al., 2000). SIMs, primarily composed of four hydrophobic amino acid residues, recognize and bind SUMOylated proteins to regulate the function and stability of substrates (Lascorz et al., 2022).

Distinct from ubiquitination, the E2 enzyme Ubc9 in SUMOylation can directly engage in substrate recognition through non-covalent interaction with SUMO molecules (Jaber et al., 2009) and may bypass E3 ligases under specific conditions (Talamillo et al., 2020; Koidl et al., 2016). Mammals encode five SUMO paralogs (SUMO1-5) that exhibit antagonistic roles in certain biological processes (Levitskaya et al., 1995; Leight and Sugden, 2000; Falk et al., 1995; Dergai et al., 2013; Zeng et al., 2020). For instance, SUMO-1 enhances transcriptional activity by promoting the interaction between specificity protein-1 (Sp1) and histone acetyltransferase p300, whereas SUMO-2 disrupts this complex and destabilizes Sp1 to exert negative regulation (Gong et al., 2014). This paralog-dependent bidirectional regulatory paradigm not only expands the versatility of SUMOylation in gene expression control but also establishes a highly plastic host-virus interface through dynamic modulation of protein stability and interaction networks during viral infection.

### 2.2 SUMOylation enhances host antiviral capacity and maintains cellular homeostasis

During HIV-1 infection, SUMOylation of host restriction factors (TRIM5α, SAMHD1) enhances host antiviral capacity by increasing their protein stability and recruiting effector proteins via SIMs. Simultaneously, SUMO modifications contribute to maintaining cellular homeostasis through dynamic regulation of stress response pathways.

Host restriction factors serve as the primary defense against HIV-1 by targeting critical steps in the viral life cycle. Recent studies demonstrate that SUMOylation and SIMs orchestrate dual mechanisms to significantly enhance the antiviral potency of multiple host factors. For instance, the host factor TRIM5α inhibits cross-species transmission by recognizing the HIV-1 capsid protein (CA) and mediating ubiquitination-dependent degradation (Ganser-Pornillos and Pornillos, 2019; Brandariz-Nuñez et al., 2013). Mechanistically, SUMOylation of TRIM5α reduces autoubiquitination-mediated degradation while modulating its E3 ligase activity, thereby promoting TRIM5α-driven activation of NF-κB and AP-1 to trigger innate immune responses (Nepveu-Traversy and Berthoux, 2014). In addition, SUMOylation mediates nuclear translocation of TRIM5α in dendritic cells (DCs), facilitating its enrichment in nucleoli and Cajal bodies to amplify antiviral efficacy (Portilho et al., 2016). Another restriction factor, SAMHD1, suppresses HIV-1 replication by depleting dNTP pools (Laguette et al., 2011; Hrecka et al., 2011), with its activity regulated by PTMs. Phosphorylation at T592 residue of SAMHD1 markedly diminishes its antiviral capacity, whereas SUMOylation at K595 and T592 dephosphorylation restores SAMHD1 activity (Martinat et al., 2021).

Beyond covalent modification, the antiviral potency of numerous host factors critically depends on SIM-mediated interactions with SUMOylated proteins. TRIM5α harbors three SIM domains (SIM1-3) (Dutrieux et al., 2015b). It has been demonstrated that SUMO1 overexpression significantly enhances anti-HIV activity of TRIM5α, whereas knockdown of SUMO1 or E2 Ubc9 abolishes this function. Intriguingly, the TRIM5α K10R substitution, deficient in being SUMOylated, does not compromise its antiviral potency (Dutrieux et al., 2015b), suggesting its activity primarily relies on SIM-dependent protein interaction networks rather than direct covalent modification. A parallel mechanism operates in SAMHD1, which possesses three SIM domains (SIM1-3). Structural analysis reveals that SIM2 integrity is indispensable for SAMHD1’s anti-HIV-1 activity, likely through recruiting host or viral cofactors to assemble functional antiviral complexes (Martinat et al., 2021). Besides, the death domain-associated protein (Daxx), a novel antiretroviral factor, inhibits HIV-1 reverse transcription predominantly via its C-terminal SIM domain despite being susceptible to SUMOylation (Dutrieux et al., 2015a). Daxx employs SIM-dependent binding to cyclophilin A (CypA) and CA proteins, thereby orchestrating nuclear recruitment of TNPO3, TRIM5α, TRIM34, and other potential cofactors. This recruitment enhances complex stability to block HIV-1 uncoating and reverse transcription (Maillet et al., 2020).

PLK1 (Polo-like kinase 1), a core serine/threonine protein kinase governing cell cycle regulation, plays essential roles in maintaining CD4^+^ T cell homeostasis (Zhou et al., 2020). HIV-1 infection induces SUMOylation of PLK1, which enhances its protein stability by antagonizing Ub-proteasome mediated degradation. The SUMO-modified PLK1 exhibits specific nuclear accumulation, and this aberrant nuclear localization activates downstream survival signaling pathways to block programmed cell death in infected cells. This pro-survival mechanism creates a critical microenvironment for establishing HIV-1 latency, thereby contributing to viral reservoir formation (Zhou et al., 2020).

### 2.3 SUMOylation-mediated host antagonism of HIV-1

The transcriptional regulation of HIV-1 primarily relies on its long terminal repeat (LTR). HIV-1 Tat protein binds to the transactivation response (TAR) RNA element within the LTR and recruits the host cyclin-dependent kinase 9 (CDK9)-cyclin T1 (CycT1) complex (positive transcription elongation factor b, p-TEFb) to activate RNA polymerase II (RNAPII)-mediated transcriptional elongation (Ali et al., 2016). The LTR region harbors binding sites for multiple transcription factors and co-regulators, forming a dynamic regulatory network to achieve high-efficiency viral transcription (El Kharroubi et al., 1998). Mounting evidence indicates that SUMOylation modulates HIV-1 transcription through multilayered regulatory mechanisms.

At the transcription factor level, STAT5 (Signal Transducer and Activator of Transcription 5), a key regulator of the JAK-STAT signaling pathway, is critically involved in HIV-1 reactivation (Dempsey, 2023). Upon stimulation by cytokines such as IL-2, STAT5 undergoes JAK-mediated tyrosine phosphorylation, leading to dimerization and nuclear translocation (Dempsey, 2023). The phosphorylated STAT5 binds to the HIV-1 LTR to promote viral reactivation (Crotti et al., 2007; Selliah et al., 2006). SUMOylation suppresses STAT5 transcriptional activity (Van Nguyen et al., 2012), while latency-reversing agents (LRAs) like benzotriazoles inhibit STAT5 SUMOylation, thereby enhancing its phosphorylation and nuclear accumulation to significantly boost viral promoter activity (Sorensen et al., 2020; Bosque et al., 2017). Within the NF-κB pathway, IκBα inhibits NF-κB nuclear translocation by binding to NF-κB dimers (Arenzana-Seisdedos et al., 1995). Competitive regulation occurs at IκBα K21, where SUMOylation stabilizes the protein by blocking ubiquitination-mediated degradation, thereby suppressing NF-κB-dependent HIV-1 gene expression (Desterro et al., 1998). Additionally, CTIP2, a chromatin-modifying complex recruitment factor at the HIV-1 LTR (Zhang et al., 2012), undergoes phosphorylation-activated SUMOylation at K679/K877, triggering proteasomal degradation and relieving transcriptional suppression (Imbert and Langford, 2021). It is worthy noted that the SUMO E3 ligase TRIM28 is recruited to the LTR during HIV-1 latency. TRIM28-mediated SUMOylation of CDK9 at K44/K56/K68 residues inhibits its kinase activity, disrupts CDK9-Cyclin T1 complex assembly, and inactivates p-TEFb to suppress transcriptional elongation (Ma et al., 2019).

At the epigenetic regulation level, Suv39h1 (a histone methyltransferase) interacts with the SUMOylation E2 enzyme Ubc9 to catalyze SUMOylation of heterochromatin protein 1α (HP1α). This modification enhances HP1α stability at heterochromatic regions, thereby maintaining LTR silencing (Maison et al., 2016). Additionally, the SUMO E3 ligase CBX4 recruits EZH2 to the HIV-1 LTR and catalyzes its SUMOylation, which reinforces EZH2-mediated deposition of repressive H3K27me3 histone marks to promote viral latency (Wu et al., 2022). Notably, the SMC5/6 complex directly modifies unintegrated HIV-1 DNA through its SUMO E3 ligase subunit NSMCE2, inducing epigenetic silencing (Irwan et al., 2022). It has been demonstrated that either TAK-981 (a SUMOylation inhibitor) or NSMCE2 loss-of-function mutations abolish this silencing effect, thereby promoting viral replication (Irwan et al., 2022). In addition, promyelocytic leukemia (PML) nuclear bodies (NBs) exhibit hyper-SUMOylation, a hallmark of aberrantly elevated SUMO modification levels. These SUMO-enriched PML NBs enhance structural stability and facilitate interactions with transcriptional repressors to cooperatively sustain viral latency (Turelli et al., 2001; Dutrieux et al., 2015a; Kahle et al., 2015; Shytaj et al., 2020).

### 2.4 SUMOylation-driven viral evasion of host defenses

In the arms race between HIV-1 and host, SUMOylation serves as a defensive strategy deployed by the host to counteract viral infection, while HIV-1 also retaliates through modulation of modification sites.

The HIV-1 Gag p6 protein, a key budding factor (Göttlinger et al., 1991), exhibits competitive interplay between SUMO-1 and Ubiquitination at K27. Monoubiquitination at K27 enhances p6 interaction with TSG101 to promote viral budding efficiency (Gurer et al., 2005). Conversely, Ubc9-mediated K27 SUMOylation markedly reduces HIV-1 infectivity, though the K27R mutation does not compromise viral replication capacity (Gurer et al., 2005). These findings underscore a complex balance between ubiquitination and SUMOylation in regulating p6 functionality.

During viral integration, SUMOylation of HIV-1 integrase (IN) at lysine residues K46/K136/K244 is critical for early replication stages, that SUMOylation-deficient mutants exhibit post-reverse transcription defects despite retaining catalytic activity and LEDGF/p75 cofactor interactions (Shinohara et al., 2002; Turlure et al., 2006; Zamborlini et al., 2011). Intriguingly, IN SUMOylation occurs not during synthesis but in subsequent infection cycles, particularly during reverse transcription and nuclear import of the preintegration complex (PIC) (Zheng and Yao, 2013). This temporal pattern suggests SUMOylation may facilitate PIC nuclear entry by modulating IN’s affinity for nuclear transport factors. Notably, IN harbors two functional SIM domains: SIM2 (200IVDI204) and SIM3 (257IKVV260) (Cherepanov et al., 2004; Shun et al., 2007), which mediate SUMO-2/3 binding. Mutations in these domains severely impair viral infectivity, reverse transcription, and integration efficiency (Zheng et al., 2019). Furthermore, Ubc9 overexpression suppresses genomic integration by enhancing IN SUMOylation, with SUMO-1/2 upregulation potentiating this inhibitory effect (Li et al., 2012). Paradoxically, SUMOylation at K364 of LEDGF/p75, while dispensable for IN binding, critically regulates reverse transcription and integration kinetics, indicating that LEDGF/p75 SUMOylation is essential for efficient HIV-1 replication (Bueno et al., 2010).

Emerging evidence suggests functional modulation of HIV-1 Rev by SUMOylation. Specifically, Rev-mediated viral replication is suppressed upon its interaction with the SUMO-1 hexapeptide repeat (SHPR) domain (Roisin et al., 2004). The viral accessory protein Vpu subverts antiviral defenses by targeting the RanBP2/RanGAP1-SUMO1/Ubc9 E3 ligase complex. This manipulation alters SUMOylation states of PML NBs and DNA repair factors, facilitating immune evasion through nuclear clearance of antiviral signaling complexes and residual viral DNA (Volcic et al., 2020).

**Figure 2:**
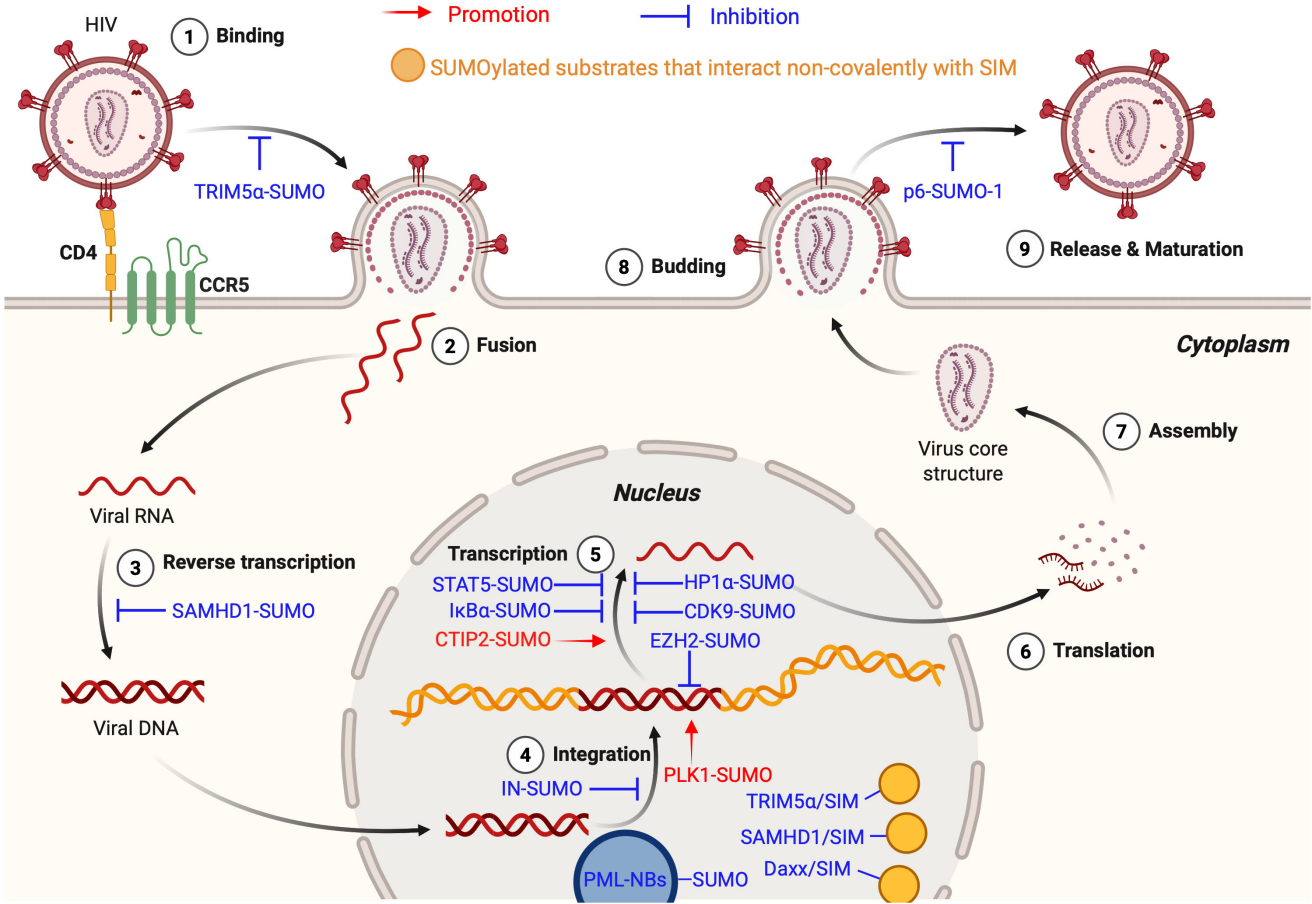
SUMOylation in the HIV life cycle. The figure illustrates the dual roles of SUMOylation in the HIV-1 life cycle, encompassing both HIV-promoting (shown in red text and arrows) and anti-HIV (shown in blue text and inhibitory symbols) effects. Yellow circles denote SUMOylated substrates that interact non-covalently with SIM. During the Binding Stage, SUMOylated TRIM5α recognizes the CA, preventing cross-species transmission. In the Reverse Transcription Stage, SUMOylated SAMHD1 inhibits replication by depleting the intracellular dNTP pool. At the Integration Stage, SUMOylated IN impairs integration, SUMOylated PLK1 supports latency maintenance, and SUMOylated EZH2 promotes epigenetic silencing. At the Transcription Stage, SUMOylated STAT5, IκBα, HP1α, and CDK9 suppress HIV-1 transcription, whereas SUMOylated CTIP2 enhances it. During Budding Stage, SUMOylated p6 reduces viral infectivity. In the nucleus, hyper-SUMOylated PML NBs facilitate the establishment and maintenance of viral latency. Additionally, SUMOylated substrates interact non-covalently with SIMs on TRIM5α, SAMHD1, and Daxx, contributing to inhibition of HIV-1 replication. This figure is generated using BioRender (http://biorender.com/).

Collectively, these findings reveal the dual roles of SUMOylation in HIV-1 infection. On one hand, SUMOylation enhances host antiviral defenses by stabilizing restriction factors (e.g., TRIM5α, SAMHD1) and organizing SIM-mediated protein interaction networks. On the other hand, HIV-1 subverts the SUMOylation machinery to promote its own replication and persistence (e.g., CTIP2, PLK1). These opposing roles highlight SUMOylation as a dynamic regulatory node in the virus-host interplay, offering both opportunities and challenges for therapeutic intervention (**Figure 2**).

## 3 ISGylation

### 3.1 Introduction of ISGylation

ISG15, a protein encoded by the interferon-stimulated gene *ISG15*, exerts multifaceted regulatory roles in antiviral immunity. The precursor of ISG15, Pro-ISG15 (~17 kDa), undergoes proteolytic processing to generate mature ISG15 (~15 kDa) (Potter et al., 1999). Although ISG15 exhibits low sequence conservation across species (e.g., 66% homology between human and murine orthologs) (Dzimianski et al., 2019), its C-terminal diglycine motif, the catalytic domain mediating ISGylation, remains highly conserved. The ISGylation cascade requires sequential action of E1 (UBE1L), E2 (UBE2L6), and E3 ligases (HERC5, TRIM25) to conjugate ISG15 to specific lysine residues on target proteins. Notably, ISG15 operates through three functional modalities: 1) intracellular free form, 2) covalent conjugates with substrates, and 3) secreted soluble form (Lenschow, 2010). Distinct from K48-linked ubiquitination that triggers proteasomal degradation, ISGylation exerts antiviral effects via non-degradative mechanisms. These include modulating viral protein subcellular localization (e.g., restricting virion assembly) and stabilizing host antiviral proteins to amplify innate immune responses (Woods et al., 2014). It is reported that ISGylation-mediated cellular outcomes depend on both infection stage and substrate specificity (Mathieu et al., 2021; Liu et al., 2003). For instance, late-stage infection may undergo ISGylation-mediated functional inhibition or degradation of host proteins, attenuating cellular immunity (Minakawa et al., 2008).

### 3.2 ISGylation inhibits HIV-1 replication

**Figure 3:**
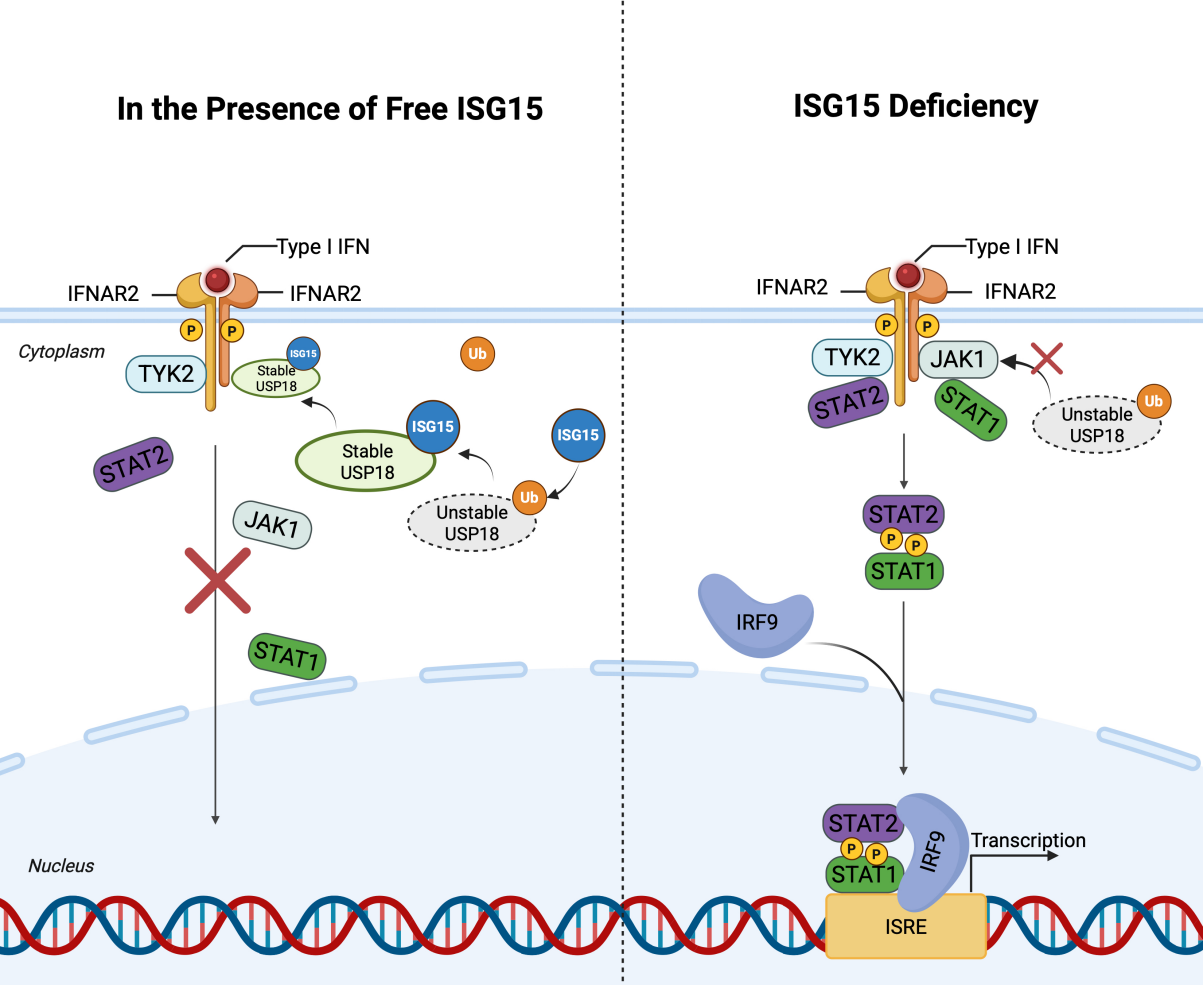
ISG15-Mediated stabilization of USP18 and negative regulation of IFN-I signaling. This figure illustrates the the interplay between ISG15 and USP18 in regulating the IFN-I signaling. Left panel: In the presence of free ISG15, USP18 is stabilized by competing with ubiquitin, thereby enhancing its association with IFNAR2 and preventing JAK1 binding. This inhibits activation of the JAK-STAT pathway and downregulating ISG expression. Right panel: In the absence of ISG15, USP18 becomes unstable, leading to sustained IFN-I signaling. Upon IFN-I binding to its receptor complex, JAK1 and TYK2 are activated, resulting in the formation of the ISGF3 complex (STAT1, STAT2, and IRF9). ISGF3 translocates into the nucleus and binds to ISREs in the promoters of ISGs. This figure is generated using BioRender (http://biorender.com/).

ISG15 functions as a host factor against HIV-1, exerting multidimensional antiviral effects through ISGylation. As a key antiviral factor, p53 inhibits HIV replication and induces apoptosis of infected cells, thereby limiting HIV-1 propagations (Xia and Jiang, 2024). Osei Kuffour et al. found that ISGylation suppresses HIV-1 replication via dual regulation of p53: stabilizing p53 by counteracting ubiquitination-mediated degradation while promoting misfolded p53 degradation (Osei Kuffour et al., 2019). Overexpression of ISG15 or its E1 enzyme UBE1L significantly inhibits both early integration and late budding stages of HIV-1 (Shirazi and Pitha, 1992; Pincetic et al., 2010; Okumura et al., 2006). Mechanistically, ISG15 disrupts Ub-dependent interaction between HIV-1 Gag and Tsg101 via ISGylation of cellular targets, thereby blocking virion release (Gómez et al., 2020; Pincetic et al., 2010). The E3 ligase HERC5 employs two distinct antiviral mechanisms: ISGylation-mediated modification of Gag to impair viral assembly at the plasma membrane and RCC1-like domain-dependent suppression of Rev/RRE-mediated RNA nuclear export (Dastur et al., 2006). Notably, HERC5’s antiviral activity is also regulated by its own ISGylation, suggesting an autoregulatory loop (Woods et al., 2014). In addition, while early studies indicated ISG15 deficiency inhibits HIV-1 infection (Bosque and Planelles, 2009), subsequent mechanistic analyses revealed that this is due to free cellular ISG15 aids the stabilization of the ubiquitin-specific peptidase 18 (USP18), a negative regulator of type I interferon (IFN-I) signaling. USP18 binds to the IFN-I receptor subunit IFNAR2 and completes with JAK1 (Speer et al., 2016), thereby attenuating JAK-STAT pathway activation. Consequently, ISG15 deficiency leads to USP18 destabilization, resulting in sustained IFN-I signaling and enhanced expression of interferon-stimulated genes (*ISGs*) (Zhang et al., 2015), which can exert antiviral effects. This explains the paradoxical observation that loss of ISG15, while eliminating its direct antiviral functions via ISGylation, can indirectly inhibit HIV-1 replication by amplifying the IFN-I response and promoting an antiviral state. These findings highlight the conflicting role of ISG15 in host-virus interactions (**Figure 3**).

These findings not only unravel the multifaceted regulatory networks of ISGylation throughout the HIV-1 life cycle but also highlight the therapeutic potential of targeting HERC5’s autoregulatory ISGylation for precise modulation of antiviral activity.

### 3.3 Immune evasion mechanism of HIV-1 via Vpu-mediated regulation of ISGylation

HIV-1 employs its viral accessory protein Vpu to establish an immune evasion that selectively targets core components of the host ISGylation system, thereby sustaining infection advantage. Okumura et al. reported that Vpu suppresses ISGylation through diverse regulatory strategy: (1) degrading the ISG15-conjugating enzyme UBE2L2 via a Ub-proteasome-independent pathway, (2) impairing the functional activity of UBE2L6, and (3) modulating subcellular distribution of the transmembrane protein PLP2. The non-canonical degradation of UBE2L2 directly disrupts ISG15-substrate binding capacity, resulting in downregulation of intracellular ISGylation (Jain et al., 2018; Okumura et al., 2006). This unique immune evasion strategy effectively compromises ISGylation-dependent antiviral responses, thereby promoting viral replication and accelerating infection-associated disease progression.

## 4 NEDDylation

### 4.1 Introduction of NEDDylation

NEDDylation is a reversible PTM mediated by the UBL molecule NEDD8 through an enzymatic cascade involving E1 (NAE, NEDD8-activating enzyme), E2 (UBE2M/UBE2F), and E3 ligases (Rbx1/Rbx2). This process regulates substrate function through covalent conjugation to lysine residues (Kamitani et al., 1997; Xirodimas et al., 2004; Xirodimas, 2008). The most well-characterized targets of NEDDylation are Cullin family proteins. All Cullin-associated ubiquitin ligases require NEDDylation for functional activation (Embade et al., 2012; Enchev et al., 2015). NEDDylation induces conformational changes in Cullins to expose substrate-binding sites, thereby activating Cullin-RING E3 ubiquitin ligase complexes (CRLs) and enhancing their ubiquitination efficiency. It has been reported that substrate specificity exists within this system: UBE2M exclusively mediates NEDDylation of Cullin1-4, while UBE2F specifically modifies Cullin5.

### 4.2 HIV exploits NEDDylation to degrade antiviral factors and enhance infection

Emerging studies reveal that HIV exploits NEDDylation-mediated ubiquitination pathways to potentiate its infectious capacity (Nekorchuk et al., 2013). Specifically, HIV hijacks host CRL complex to degrade antiviral restriction factors. In HIV-2 infection, the viral accessory protein Vpx, via its evolutionarily conserved HHCC motif and zinc-binding domain (Guo et al., 2019; Wang et al., 2017), interacts with the CRL4 complex (comprising DDB1, RBX1, Cullin4A, and DCAF1) to induce NEDDylation of the Cullin4 subunit. This covalent NEDD8 modification activates CRL4’s E3 ubiquitin ligase activity, driving K48-linked ubiquitination and subsequent proteasomal degradation of SAMHD1 (Hrecka et al., 2011; Laguette et al., 2011; Hofmann et al., 2013).

The host restriction factor APOBEC3G exerts antiviral effects by deaminating cytosine (C) to uracil (U) in viral single-stranded DNA during reverse transcription, impairing viral genome integrity and progeny infectivity (Iwatani et al., 2007; Bishop et al., 2006). Counteractively, HIV-1 Vif recruits UBE2F to mediate Cullin5 NEDDylation, activating the CRL5Vif-CBFβ complex. This machinery catalyzes K48 ubiquitination of APOBEC3G, marking it for proteasomal destruction (Yu et al., 2003; Liu et al., 2005; Stanley et al., 2012).

Furthermore, HIV-1 Vpr targets uracil-N-glycosylase (UNG2), which exhibits dual regulatory roles in HIV-1 replication: on one hand, it enhances the fidelity of reverse transcription (Chen et al., 2004); on the other hand, its nuclear-localized isoform participates in cDNA degradation and maintains host genomic stability by repairing uracil mismatches (Akbari et al., 2004). Notably, the HIV-1 viral protein Vpr targets UNG2 to promote NEDD8 modification of the CRL4 complex, thereby inducing CRL4-mediated ubiquitination and degradation of UNG2 (Nekorchuk et al., 2013). This mechanism not only compromises host DNA repair capacity but also enhances HIV-1 replication, thereby promoting viral dissemination.

In summary, host CRL complexes collaborate with viral proteins to orchestrate ubiquitination-dependent elimination of antiviral factors. Given that CRL activity strictly depends on NEDD8 modification of conserved lysine residues within the C-terminal domains of Cullin proteins, this PTM regulatory strategy becomes indispensable for HIV infection. These findings not only elucidate novel viral strategies to subvert immune surveillance but also provide a mechanistic foundation for developing NEDDylation-targeted antiviral therapies.

### 4.3 NEDDylation inhibitors block HIV infection

Given that HIV and other viruses exploit NEDDylation to enhance infectivity (Abounouh et al., 2022; Lee et al., 2022; Zhang et al., 2021; Nekorchuk et al., 2013), inhibition of this modification has emerged as a promising therapeutic strategy. The NEDD8-activating enzyme (NAE) inhibitor MLN4924, a broad-spectrum antiviral candidate, specifically blocks NEDD8 conjugation to CRLs, effectively perturbing ubiquitination cascades (Xie et al., 2021; Xu et al., 2018). Recent studies demonstrate that MLN4924 suppresses Cullin-mediated degradation pathways, thereby counteracting HIV accessory protein functions, including Vif-driven APOBEC3G elimination, Vpr-mediated UNG2 depletion, and Vpx-dependent SAMHD1 degradation (Stanley et al., 2012; Nekorchuk et al., 2013; Wei et al., 2014).

The antiparasitic agent suramin has garnered significant attention for its potential to disrupt pathogen proliferation (Steverding and Troeberg, 2023). Recent investigations identify suramin as a novel HIV inhibitor that synergizes with MLN4924 to enhance therapeutic efficacy (Zhang et al., 2020). Mechanistically, suramin interferes with biosynthesis of inositol hexakisphosphate (IP6), a critical cofactor for viral capsid assembly. Suramin and its analog NF449 suppress IP6 production by targeting IP5 kinase (IP5K), inhibiting ATP-binding activity and IP5 phosphorylation. Depleted IP6 levels impair COP9 signalosome (a CRL-deneddylation enzyme complex) recruitment to CRLs, thereby disrupting the dynamic equilibrium required for CRL activity cycling (Dick et al., 2018; Mallery et al., 2019; Zhang et al., 2020). This regulatory mechanism, simultaneously inhibiting CRL activation via NEDDylation blockade and preventing CRL inactivation through deneddylation impairment, positions the combinatorial regimen as a promising approach for achieving comprehensive suppression of HIV replication.

Although NEDDylation inhibitors hold promise as antiviral agents, their clinical translation is impeded by substantial challenges. MLN4924 is currently in phase II clinical trials for various hematological malignancies and solid tumors (Zhou et al., 2018). In addition to its antitumor activity, studies have demonstrated that MLN4924 exhibits significant antiviral effects against a broad spectrum of viruses, including Human Cytomegalovirus (HCMV), Herpes simplex virus (HSV)-1, HSV-2, adenovirus 5 (AdV5), influenza virus PR8 and HIV (Le-Trilling et al., 2016). However, there remains a paucity of clinical studies to substantiate its anti-virus applications. Suramin, initially developed for parasitic infections, has also demonstrated anti-HIV properties and can synergize with MLN4924 to enhance therapeutic efficacy. However, suramin’s low therapeutic index and frequent off-target effects, attributable to its interaction with multiple cellular targets, pose substantial limitations for clinical use (Guo et al., 2021a). Moreover, given that NEDDylation is essential for maintaining normal cellular homeostasis, systemic inhibition may lead to concerns regarding toxicity and specificity. To address these challenges, future strategies should focus on enhancing selectivity, such as by targeting virus-specific NEDDylation events or employing cell-type-specific delivery systems, thereby minimizing host toxicity while maximize therapeutic efficacy.

## 5 ATG8ylation

### 5.1 Introduction of ATG8ylation

ATG8ylation, a PTM closely associated with autophagy, involves the synthesis of its core molecule ATG8 as an inactive precursor containing a C-terminal non-functional amino acid extension. This modification is initiated by E1 enzyme ATG7-mediated proteolytic processing of the ATG8 precursor, which cleaves the C-terminal extension to expose a glycine residue, generating the reactive mature ATG8. Subsequently, through the coordinated action of E2 (ATG3) and E3 (ATG12), mature ATG8 covalently conjugates to substrate proteins or membranes. Notably, the ATG8 family comprises multiple homologs, with LC3 and GABARAPL2 being the predominant forms in humans (Kumar et al., 2020b).

### 5.2 Host maintains homeostasis through the autophagy pathway

The tripartite motif (TRIM) protein family serves as critical host antiviral factors by modulating autophagy pathways. Research demonstrates that TRIM5 overexpression significantly activates cellular autophagy processes (Mandell et al., 2014). Furthermore, TRIM5 functions as a selective autophagy receptor through direct interaction with core autophagy proteins of the ATG8 family, particularly GABARAP subtypes, enabling specific recognition of retroviral particles. Notably, this recognition mechanism operates independently of the canonical ubiquitination system. All TRIM family members directly interact with at least one GABARAP subtype, establishing a universal interaction paradigm. This unique molecular strategy allows TRIM5 to direct autophagosomes toward HIV-1 viral particles for degradation, thereby effectively protecting host cells from viral infection (Mandell et al., 2014). These findings emphasize the critical role of GABARAP, rather than LC3, in mediating selective autophagic immune responses against HIV-1.

### 5.3 HIV-1 enhances infection through ATG8ylation

ATG8ylation exerts multidimensional regulatory effects throughout the HIV-1 infection cycle. During viral entry, this modification enhances infectivity primarily by facilitating membrane fusion between the virus and host. Specifically, upon HIV-1 exposure to CD4^+^ T lymphocytes, the ATG8 family member LC3B rapidly accumulates at the plasma membrane, even before direct virion contact, suggesting a priming role for membrane remodeling. This recruitment is dependent on Env-CD4 binding and requires key ATG8ylation enzymes such as ATG7 and BECN1, but notably bypasses canonical autophagy initiation factors like ATG13. Such findings demonstrate that LC3B lipidation (i.e., ATG8ylation) serves as a distinct, autophagy-independent mechanism facilitating the membrane curvature or fluidity changes required for viral fusion. Thus, LC3-mediated membrane dynamics serve as a critical molecular switch for HIV-1 entry, although further studies are needed to delineate its coordination with cytoskeletal remodeling and lipid signaling pathways (Pradel et al., 2024).

Furthermore, during HIV-1 infection, mammalian ATG8 homologs (mAtg8s), particularly GABARAP, orchestrate a finely tuned regulatory network involving lysosomal biogenesis and immune restriction. Mechanistically, GABARAP directly binds TFEB and facilitates its nuclear translocation independent of canonical LIR-LDS interactions. This process is modulated by IRGM, which promotes TFEB dephosphorylation both by inhibiting mTOR and activating the phosphatase PPP3CB. Knockout studies further demonstrate that GABARAPs, but not LC3s, are indispensable for starvation- or stress-induced TFEB activation and downstream lysosomal gene expression. In the context of HIV-1, GABARAP also interacts with TRIM5, a cytosolic restriction factor that recognizes the viral capsid and recruits the autophagic machinery to degrade it. These dual functions, enhancing lysosomal capacity via TFEB and mediating innate immune recognition via TRIM5, highlight GABARAP as a key node bridging autophagy, immune defense, and viral modulation (Kumar et al., 2020a).

Notably, HIV-1 Nef protein hijacks the IRGM-TFEB pathway to suppress lysosomal degradation of viral components, establishing a unique “virus-protective autophagic inhibition” mechanism (Kyei et al., 2009). This strategy prevents HIV components from lysosomal destruction, effectively shielding the virus from autophagic clearance during host cell defense. Saribas et al. reported that Nef induces aberrant accumulation of ATG8/LC3 and p62 (SQSTM1), mimicking the action of the autophagy inhibitor Bafilomycin A1 (BAFA1). By blocking autophagosome-lysosome fusion, Nef enables HIV-1 to evade autophagic elimination in human astrocytes (Saribas et al., 2015).

During viral release, HIV-1 Vpu exploits its L_63_VEM_66_ motif to specifically bind the ATG8 family member LC3C. In concert with ATG6 (Beclin1) and LC3C, this interaction enhances Vpu’s ability to counteract BST2-mediated restriction (Madjo et al., 2016). These findings collectively demonstrate that the ATG8ylation system not only modulates viral entry but also facilitates virion maturation and release by remodeling host membrane dynamics.

Collectively, these findings demonstrate that the ATG8ylation system involves distinct functions of LC3 and GABARAP subfamilies, with LC3 primarily mediating membrane fusion events facilitating viral entry and release, while GABARAP mainly governs selective autophagic immune responses and lysosomal regulation, highlighting the dual roles within the ATG8 family in HIV-1 regulation.

## 6 FAT10ylation

### 6.1 Introduction of FAT10ylation

FAT10, a multifunctional UBL modifier, serves dual roles in cellular immune surveillance and dynamic protein homeostasis by mediating targeted degradation of substrates (Raasi et al., 1999; Mah et al., 2019). The FAT10ylation process involves covalent conjugation of FAT10 to substrates or membranes through a cascade comprising the E1 (UBA6), E2 (USE1), and putative E3 ligases. Unlike canonical ubiquitin and other UBLs, however, FAT10 appears to function with minimal or undefined involvement of dedicated E3 ligases. To date, no FAT10-specific E3 ligase has been conclusively identified, suggesting a potentially non-canonical or more substrate-intrinsic mechanism of substrate selection and modification (Chiu et al., 2007; Aichem et al., 2010, 2014). This apparent absence represents a major gap in our understanding of the FAT10ylation machinery and raises questions about how substrate specificity and regulation are achieved in different cellular contexts. Another unique feature of FAT10 is its C-terminal diglycine motif, which, although functionally analogous to that of ubiquitin, is inherently exposed in its primary sequence and does not require proteolytic processing to achieve conjugation competence. This contrasts with ubiquitin and several other UBLs that are synthesized as precursors requiring cleavage to expose the C-terminal Gly-Gly motif. Despite increasing interest in FAT10 biology, many aspects of its substrate landscape remain obscure. In particular, the identities and functional consequences of FAT10-specific substrates in the context of HIV-1 infection remain largely uncharacterized. Given the virus’s dependence on host proteostasis and immune modulation, elucidating how FAT10-mediated degradation affects viral replication or host antiviral responses could uncover novel therapeutic opportunities. Further studies are urgently required to define substrate repertoires, ascertain the existence of virus-encoded regulators of FAT10ylation, and characterize how HIV-1 might hijack or evade this PTM system.

### 6.2 HIV-1 induces FAT10 overexpression to promote apoptosis

FAT10ylation exerts multidimensional biological effects within the HIV-1 infection. In HIV-associated nephropathy (HIVAN) models, studies have delineated that FAT10ylation is involved in disease progression through pro-apoptotic pathways: HIV-1-infected renal tubular epithelial cells (RTECs) exhibit significant FAT10 upregulation, which activates intrinsic apoptotic cascades to drive RTEC apoptosis (Ross et al., 2006). RNA interference-mediated silencing of endogenous FAT10 expression effectively abrogates HIV-1-induced RTEC apoptosis, confirming FAT10’s central regulatory role in this pathological cascade (Ross et al., 2006).

HIV-1 Vpr has been identified as a critical inducer of FAT10 expression in RTECs, promoting apoptosis by elevating FAT10 levels in these cells (Snyder et al., 2009). Snyder et al. found that FAT10 directly interacts with Vpr and serves as a key mediator of Vpr-driven RTEC apoptosis. However, the precise mechanism by which FAT10 facilitates Vpr-induced apoptosis remains unresolved. Furthermore, it remains unclear whether FAT10-mediated apoptosis is pathogenic to the host or represents an adaptive antiviral response to limit viral spread through covalent modification of target proteins (Snyder et al., 2009).

Similar to ubiquitination, FAT10ylation can trigger substrate degradation (Yi et al., 2020). Emerging evidence suggests FAT10ylation may inhibit HIV-1 by degrading viral proteins essential for infection (Kubo et al., 2022). Nevertheless, the mechanistic basis of FAT10ylation’s antiretroviral activity requires further investigation.

## 7 Therapeutic targeting of UBL pathways: opportunities and challenges

The maintenance of intracellular protein homeostasis is essential for organismal health, and its disruption has been implicated in numerous pathological conditions (Dowell et al., 2007). The ubiquitin–proteasome system (UPS) plays a central role in regulating protein degradation, where the 26S proteasome recognizes and hydrolyzes polyubiquitinated proteins to control various cellular processes (Coll-Martínez and Crosas, 2019). Many viruses, including HIV, exploit the UPS pathway to regulate the stability of their own proteins or to induce the degradation of host restriction factors, therefore enhancing viral replication (Barry and Früh, 2006; Gustin et al., 2011). Thus, inhibition of the UPS pathway is an effective strategy to suppress viral replication. Compounds targeting the ubiquitin-conjugation system (e.g., PYR-41) and the proteasome (e.g., MG-132, Bortezomib) have been shown to interfere with viral replication (Kaspari et al., 2008; Luo et al., 2003; Satheshkumar et al., 2009; Schubert et al., 2000). However, the toxicity of these agents can lead to severe side effects in the host: upon proteasome inhibition, the half-life of more than 80% of cellular proteins is significantly increased (Meierhofer et al., 2008; Yen et al., 2008). So far, no clinical studies have reported the use of PYR-41, MG132, or Bortezomib in HIV therapy, highlighting the need for further investigation into their potential antiviral effects.

As outlined in Section 2.3, the anticancer drug TAK-981 is a specific inhibitor of the SUMOylation E1 enzyme (Langston et al., 2021). The SUMOylation of the SMC5/6 complex triggers epigenetic silencing of unintegrated HIV-1 DNA. TAK-981 disrupts the structural stability of PML NBs, functioning as the “shock” component in the “shock and kill” strategy, and demonstrating potential therapeutic value in HIV treatment (Irwan et al., 2022). Although TAK-981 has entered Phase I clinical trials for the treatment of solid tumors and lymphomas (Lightcap et al., 2021), there are no clinical reports available concerning its application in HIV treatment.

What’s more, as mentioned in Section 4.3, MLN4924 is a selective inhibitor of NAE, which prevents the activation of CRLs and consequently blocks the NEDDylation process (Xie et al., 2021; Xu et al., 2018). MLN4924 has advanced to phase II clinical trials and demonstrated promising antitumor activity in preclinical and clinical settings (Zhou et al., 2018). Its toxicity profile has been extensively studied. Although some adverse effects have been observed, they are generally milder, and to date, no grade 4 adverse events and treatment-related deaths have been reported (Shah et al., 2016). However, UBL-specific inhibitors beyond the NEDDylation pathway remain largely unexplored. High-throughput virtual screening based on compound libraries, combined with structural modeling of key enzymes involved in UBL cascades, represents a viable approach for identifying promising molecules. Although such strategies may yield numerous candidate compounds, significant challenges persist in drug development. Key concerns include the efficient intracellular delivery of compounds to targets, minimizing off-target toxicity, and ensuring sufficient therapeutic efficacy. For instance, suramin has demonstrated inhibitory activity against NEDDylation, but its clinical application has been limited by considerable side effects and suboptimal efficacy (Guo et al., 2021a). Future efforts may focus on chemical modification of existing compounds or combination therapies to enhance binding affinity to UBL-specific targets while reducing adverse effects. Overcoming these pharmacological limitations will be essential for translating UBL-targeted therapies into clinical effective treatments.

## 8 Discussion

**Figure 4:**
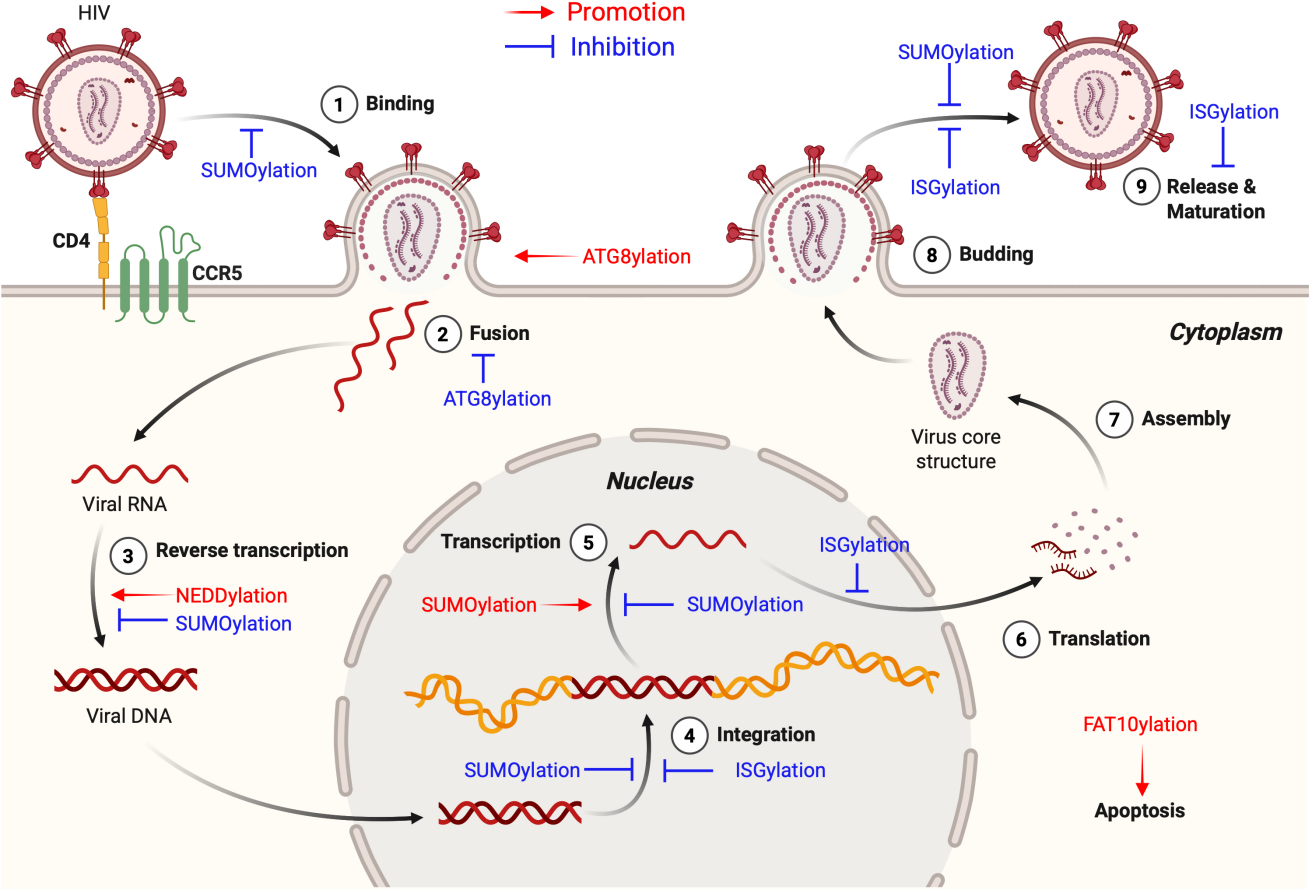
Roles of UBL modifications in the HIV-1 life cycle. This figure highlights the roles of various UBL modifications in the HIV-1 life cycle, demonstrating both HIV-promoting (shown in red text and arrows) and anti-HIV (shown in blue text and inhibitory symbols) functions. SUMOylation related regulatory mechanisms are described in detail in **Figure 2**. ISGylation stabilizes the tumor suppressor protein p53 to limit HIV-1 replication, inhibits early post-entry steps including viral DNA integration, and interferes with viral budding and release. NEDDylation promotes viral replication by facilitating the formation of CRL complexes that target host restriction factors such as A3G, SAMHD1, and UNG2 for degradation. ATG8ylation contributes to viral entry through LC3 lipidation at the plasma membrane, which drives membrane remodeling, and also mediates selective autophagic degradation of HIV-1 virions via TRIM5-GABARAP interactions. FAT10ylation plays a HIV-promoting role as the HIV-1 accessory protein Vpr induces FAT10 expression in RTECs, activating intrinsic apoptotic pathways and promoting cell death. This Figure is drawn using the BioRender website. This figure is generated using BioRender (http://biorender.com/).

HIV remains a major global health threat, with its infection process highly dependent on the precise regulation of the protein PTM system. PTMs orchestrate HIV infection by reversibly modifying both viral and host proteins, thereby altering their structure, function, and interactions to influence viral replication cycles, immune evasion, and host defense responses. Among diverse PTM types, the canonical regulatory mechanism ubiquitination governs HIV infection through substrate degradation, localization control, and signaling modulation. Recent advances have unveiled expanding roles of UBLs, including SUMOylation, ISGylation, NEDDylation, ATG8ylation, and FAT10ylation, in regulating HIV-host interactions. This review systematically summarizes the multidimensional functions of UBL networks in HIV infection, highlighting their spatiotemporal regulation of viral entry, replication, latency, and budding (**Table 1**) (**Figure 4**).The bidirectional dynamic regulation of PTM is particularly prominent in HIV infection. Deubiquitinating enzymes (DUBs), as key regulatory factors, reverse ubiquitination to participate in viral replication and serve as critical mechanisms for intracellular regulation of protein function, stability, and interactions. Different types of PTMs dynamically add or remove modifying molecules to precisely regulate protein biological functions. During HIV infection, the regulatory role of PTMs is especially crucial, particularly the impact of ubiquitination and UBL modifications on the viral life cycle (Teh et al., 2022). For example, USP21 inhibits HIV-1 replication by downregulating Tat expression (Gao et al., 2021a), while USP7 stabilizes Tat to exert the opposite effect (Ali et al., 2017). USP37 enhances SAMHD1’s anti-HIV-2/SIV activity (Cui et al., 2025), and USP8 counteracts Vif-mediated A3G degradation (Gao et al., 2021b). Notably, UBL systems also possess corresponding deconjugating enzymes (DUBLs). SUMOylation is regulated by the SENP family (Li and Hochstrasser, 1999, 2000), where SENP-mediated deSUMOylation of integrase IN significantly suppresses viral infectivity (Madu et al., 2015). Additionally, the CSN complex (containing CSN5/JAB1) promotes HIV-1 replication in CD4^+^ T cells through deneddylation (Kinoshita et al., 2012). USP18 enhances HIV-1 replication by inhibiting ISG15 synthesis (Lin et al., 2024). These studies suggest that DUBs/DUBLs constitute critical regulators in the host-virus interplay. We propose that other UBLs may harbor undiscovered DUBLs, and systematic characterization of their functional networks will provide novel targets for intervening in HIV infection.

**Table 1:** The effects of UBL modifications on host factors and HIV proteins.

Ubiquitin-like Modifications	E1	E2	E3	Effects on Host Factors	Effects on HIV Proteins
SUMOylation	SAE1/SAE2	Ubc9	PIAS Family and RanBP2 ,etc	1. Enhancing the Antiviral Activity of Host Restriction Factors TRIM5α (Dutrieux et al., 2015b; Nepveu-Traversy and Berthoux, 2014; Portilho et al., 2016), SAMHD1 (Martinat et al., 2021), Daxx (Maillet et al., 2020) 2. Maintaining PLK1 Stability to Support Cell Survival (Zhou et al., 2020) 3. SUMOylation of STAT5 Inhibits HIV Transcription (Sorensen et al., 2020; Bosque et al., 2017) 4. SUMOylation of IκBα Inhibits HIV Transcription (Desterro et al., 1998) 5. SUMOylation of CDK9 Inhibits HIV Transcription (Ma et al., 2019) 6. SUMOylation of HP1α Mediates HIV Silencing (Maison et al., 2016) 7. SUMOylation of HP1α Mediates HIV Silencing (Wu et al., 2022) 8. SUMOylation of the SMC5/6 Complex Mediates Epigenetic Silencing of HIV-1 DNA (Irwan et al., 2022) 9. Hyper-SUMOylation of PML NBs Inhibits HIV Replication (Turelli et al., 2001; Dutrieux et al., 2015a; Kahle et al., 2015; Shytaj et al., 2020)	1. SUMOylation of p6 Reduces Infectivity (Göttlinger et al., 1991; Gurer et al., 2005) 2. SUMOylation of IN Inhibits Intergration Activity (Zamborlini et al., 2011) 3.Rev binds to SHPR to mediate HIV-1 replication inhibition (Roisin et al., 2004).
ISGylation	UBE1L	UBE2L6,etc	HERC5 TRIM28	1. ISGylation of p53 Inhibits HIV-1 Replication (Osei Kuffour et al., 2019)	1. ISGylation of Gag Inhibits Early Assembly of HIV-1 (Dastur et al., 2006)
NEDDylation	NAE	UBE2M or UBE2F	Rbx1,Rbx2,etc	1. Mediating the Degradation of SAMHD1 to Enhance Infection (Hrecka et al., 2011; Laguette et al., 2011; Zhang et al., 2021; Hofmann et al., 2013) 2. Mediating the Degradation of APOBEC3G to Enhance Infection (Yu et al., 2003; Liu et al., 2005) 3. Mediating the Degradation of UNG2 to Enhance Infection (Nekorchuk et al., 2013)	Unidentified
ATG8ylation	ATG7	ATG3	ATG12-ATG5-ATG16L1 complex	1. The Interaction Between TRIM5 and GABARAP Restricts HIV-1 Infection (Mandell et al., 2014) 2. The Interaction Between LC3B and the Plasma Membrane Promotes HIV-1 Entry into Cells (Mandell et al., 2014) 3. The Interaction Between IGRM and GABARAP Promotes HIV-1 Entry into Cells (Kumar et al., 2020a)	1. The Interaction Between HIV-1 Vpu and LC3C Antagonizes the Restriction of BST2 (Madjo et al., 2016)
FAT10ylation	UBA6	USE1	Unknown	1. Apoptosis-Related Proteins Induce the Death of Infected Cells (Ross et al., 2006; Snyder et al., 2009)	Unidentified

Breakthroughs in ubiquitination research have established critical paradigms for exploring UBL mechanisms. Mabbitt et al. found that the human E3 ubiquitin ligase Myc binding protein 2 (MYCBP2) modifies threonine (Thr) residues via a “RING-Cys-relay” (RCR) mechanism, extending UBL modifications even to non-amino acid molecules such as glycerol (Mabbitt et al., 2020). Furthermore, PTMs of Ub itself, exemplified by serine 65 (Ser65) phosphorylation, dynamically regulate its functionality through multiple mechanisms: impairing E2-E3 pairing (Wauer et al., 2015), altering binding domain affinities (Swaney et al., 2015), and modulating Ub chain topology (Gersch et al., 2017). These findings illuminate potential unconventional UBL modification modes, suggesting that UBL modifiers may similarly target non-lysine residues (e.g., Ser/Thr) or non-protein substrates, while their own PTMs (e.g., phosphorylation) could spatiotemporally regulate conjugation dynamics. Systematic investigation of these atypical UBL modification paradigms will profoundly expand our understanding of multidimensional virus-host interplay.

Emerging studies have proved multilevel crosstalk among SUMOylation, ubiquitination, and ISGylation. Interferon (IFN) signaling coordinately upregulates SUMO3, ubiquitination, and ISGylation pathways by stabilizing enzymes like UBE2L6 and HERC5, thereby enhancing expression of antiviral proteins including IFITMs and SAMHD1 (Kubota et al., 2008; Nakagawa and Yokosawa, 2002; Ran et al., 2011). Key regulatory paradigms include: (1) Competitive modification, SUMOylation of TRIM5α antagonizes its ubiquitination to maintain protein stability (Nepveu-Traversy and Berthoux, 2014); (2) Functional interference, ISG15 conjugation to Ubc13 impairs its Ub-binding capacity (Takeuchi and Yokosawa, 2005; Zou et al., 2005); (3) Synergistic enhancement, SUMOylation stabilizes MDM2 to promote Ub-dependent p53 degradation (Buschmann et al., 2001), while NEDDylation activates CRLs to amplify substrate ubiquitination (Hofmann et al., 2013; Yu et al., 2003; Liu et al., 2005; Stanley et al., 2012; Nekorchuk et al., 2013). These interactions demonstrate that UBLs can independently or cooperatively regulate pivotal biological processes. Systematic elucidation of their interaction networks will provide a conceptual framework for designing multi-target therapeutic strategies.

In summary, the UBL system orchestrates HIV infection through multilayered, dynamically reversible regulatory networks. Mechanistic dissection and therapeutic exploitation of this system, particularly through discovery of novel modification types, exploration of non-canonical modification paradigms, and elucidation of DUB/DUBL regulatory circuits, hold transformative potential for developing novel strategies against HIV.
